# Long-term functional and radiological outcomes among survivors of ankylosing spondylitis-associated cervical spine fractures: a cross-sectional follow-up study

**DOI:** 10.1016/j.xnsj.2026.100908

**Published:** 2026-05-30

**Authors:** Pål Nicolay Fougner Rydning, Hege Linnerud, Vidar Stenset, Jalal Mirzamohammadi, Dag Ferner Netteland, Tor Brommeland, Magnus Evjensvold, Carl-Johan Henie Sogn, Eirik Helseth, Markus Karl Hermann Wiedmann

**Affiliations:** a Department of Neurosurgery, Oslo University Hospital, Kirkeveien 166, Oslo, 0450, Norway; b Faculty of Medicine, Institute of Clinical Medicine, University of Oslo, Postboks 1171, Blindern 0318, Oslo, Norway; c Department of Neuroradiology, Oslo University Hospital, Kirkeveien 166, Oslo, 0450, Norway

**Keywords:** Ankylosing spondylitis, Spinal fractures, Cervical vertebrae, Spinal cord injury, Patient-reported outcome, Radiological fusion

## Abstract

**Background:**

Patients with ankylosing spondylitis (AS) are predisposed to unstable cervical spine fractures, often following low-energy trauma. While short-term outcomes have been described, long-term functional and radiological outcomes among survivors remain poorly characterized.

**Methods:**

Survivor cohort study with combined retrospective and prospective case identification and cross-sectional long-term follow-up, including patients with AS-associated cervical spine fractures treated at a single regional neurotrauma center between 2010 and 2020. Surviving patients underwent cross-sectional long-term follow-up including patient-reported outcomes, neurological and functional assessment, and cervical CT imaging. Primary outcomes included Neck Disability Index, Numeric Rating Scale for neck pain, American Spinal Injury Association Impairment Scale (AIS), Karnofsky Performance Status, living status, and CT-based fracture healing.

**Results:**

Among 144 patients with AS-associated cervical spine fractures, 102 were alive and eligible for follow-up; 97 completed assessments at a median follow-up of 4.2 years. Median Neck Disability Index was 16% (IQR 6–30). Most patients lived independently (80%), with median Karnofsky Performance Status 80 (IQR 70–90). Among patients with spinal cord injury, 50% recovered to AIS E and 71% improved by at least 1 AIS grade. Neck pain was generally mild (median Numeric Rating Scale 2), and CT demonstrated solid bony fusion in 96% of assessed cases.

**Conclusions:**

Among long-term survivors, neck-related disability and pain were generally low, independence was preserved in most patients, and fracture healing rates were high.

## Highlights

• Long-term disability was generally low after ankylosed cervical spine fractures.
• Approximately half of survivors with cervical spinal cord injury improved to AIS E.
• Most surviving patients retained independent functional status.
• Long-term CT follow-up demonstrated high rates of bony fusion.

## Introduction

Patients with ankylosing spondylitis (AS, Mb Bekhterev) typically experience chronic inflammatory back and neck pain [1,2]. Progressive vertebral ankylosis and long lever arms transform the spine into a rigid, tubular structure—the so-called *“bamboo spine”*—with markedly reduced capacity to absorb external forces, rendering it highly susceptible to unstable fractures even after low-energy trauma such as ground-level falls [[3], [4], [5], [6], [7]].

Although no curative treatment exists for AS, disease-modifying antirheumatic drugs (DMARDs) provide substantial symptom relief and may slow disease progression in many patients [8,9]. Despite this, the risk of spinal fracture remains elevated and most commonly manifests in the sixth or seventh decade of life [[10], [11], [12]].

Surgical fixation is generally recommended for AS-associated cervical spine fractures because of the high instability of these injuries [[13], [14], [15]]. However, surgery is often technically demanding due to fixed spinal deformity, distorted anatomy, and a pronounced bleeding tendency [[16], [17], [18]], and is further complicated by frequent cardiovascular and pulmonary comorbidities [19,20]. Consequently, conservative management is usually reserved for frail, elderly, or highly comorbid patients, but may carry an increased risk of secondary displacement and neurological deterioration [3,4,14,[21], [22], [23]].

Beyond fracture-related morbidity, individuals with ankylosing spondylitis have higher rates of work disability and greater limitations in activities of daily living compared with the general population [24,25]. Although disease activity and function in AS are commonly assessed using composite indices, such as the Bath Ankylosing Spondylitis Disease Activity Index (BASDAI) and the Bath Ankylosing Spondylitis Functional Index (BASFI), neck-specific patient-reported outcome measures, such as the Neck Disability Index (NDI) [26], have been used to quantify cervical pain and disability in nonfracture AS cohorts. In such studies, neck-related disability is common and has been reported to increase with disease duration, while cervical spine involvement has been shown to increase with both age and disease duration [2,[27], [28], [29]]. However, data on long-term clinical and radiological outcomes following cervical spine fractures in AS—particularly with exploratory comparison between surgically and nonsurgically managed patients—remain limited.

The aim of the present study was to evaluate long-term clinical, functional, and radiological outcomes in a survivor cohort with ankylosing spondylitis treated for cervical spine fractures and to explore long-term disability for surgically and nonsurgically managed patients within a prospectively captured subcohort.

## Materials and methods

### Study design and setting

Oslo University Hospital is the sole neurotrauma center serving Southeast Norway Health Region. Trauma-related cervical spine fractures in the region are centralized to this institution. The regional population was approximately 3 million during the study period.

This study represents a survivor cohort nested within a population-based cervical spine fracture registry, including individuals with ankylosing spondylitis who sustained a traumatic cervical spine fracture between 2010 and 2020. Patients treated from 2010 to 2014 were identified retrospectively from surgical protocols, while patients treated from 2015 to 2020 were prospectively recorded in a regional quality-control registry [30].

The Regional Committee for Medical and Health Research Ethics reviewed the project and classified the study as a quality control project outside the scope of the Norwegian Health Research Act, for which formal REC approval was not required (ref. no. 2019/133). The study was approved by the Oslo University Hospital Data Protection Officer (DPO approval no. 18/24228). Informed consent was obtained from all participants in the long-term follow-up study. The regional quality control database for traumatic cervical spine fractures in Southeast Norway was also approved by the Oslo University Hospital Data Protection Officer (DPO approval no. 2014/12304). The study was conducted in accordance with the Declaration of Helsinki and reported in accordance with the STROBE guidelines.

### Patient identification and inclusion criteria

All consecutive patients with radiologically confirmed traumatic cervical spine injuries (C0/C1–C7/T1) residing within the Southeast Norway catchment area were eligible for inclusion. A diagnosis of AS was accepted when documented in the medical record by a rheumatologist or treating physician, or when characteristic imaging findings demonstrated multilevel spinal ankylosis (whole-spine) and fracture morphology consistent with AS by a consultant neuroradiologist. Patients with imaging features considered more consistent with diffuse idiopathic skeletal hyperostosis were excluded. Because some patients lacked a formal rheumatological diagnosis, the cohort therefore represents patients with AS or AS-like ankylosed spines rather than a uniformly rheumatologically verified AS cohort.

Since October 2014, all patients with cervical spine fractures, regardless of fracture type, underlying diagnosis (AS or non-AS), or management strategy, have been scheduled for standardized outpatient follow-up including clinical assessment and cervical CT imaging at 2, 6, and 12 weeks postinjury.

All identified surviving patients injured between 2010 and 2020 were contacted between 2019 and 2022 and invited to undergo clinical and radiological evaluation in an outpatient setting, conducted between 2019 and 2025. Follow-up was performed as a cross-sectional long-term assessment; however, 10 patients were assessed before 12 months, and sensitivity analyses excluding these patients were therefore performed. Follow-up duration represented the interval between the index injury and study contact, and varied between individuals accordingly. nonoperatively managed AS-associated cervical spine fractures (AS-CS-Fx) were incompletely captured prior to 2015, resulting in a predominantly surgically treated cohort during the period 2010–2014 (only 3 nonoperative cases among 44 patients). Consequently, comparative exploratory analyses between surgically and nonsurgically managed patients were restricted to the 2015–2020 period, during which prospective registration ensured complete case capture.

Baseline variables included demographic characteristics; use of antiplatelet or anticoagulant therapy (yes/no); preinjury American Society of Anesthesiologists (ASA) physical status classification [31]; preinjury living status (independent or dependent/institutionalized); preinjury occupational status; injury mechanism; fracture level; AO Spine fracture classification [32]; spinal cord injury severity graded according to the American Spinal Injury Association Impairment Scale (AIS) [33]; presence of radiculopathy (defined as dermatomal pain or numbness, yes/no); polytrauma (yes/no); treatment strategy (surgical vs. nonsurgical); and surgical construct. Polytrauma was defined as a concomitant traumatic brain injury, and/or radiologically verified injury to the face, thoracolumbar spine, chest, abdomen, pelvis or extremities. Superficial skin injuries were excluded.

### Management of fractures

Surgical treatment of AO Spine type B or C fractures was performed using either an anterior-only cervical approach (typically spanning 1–3 levels), a posterior-only approach (typically spanning 4 levels), or a combined circumferential approach. Postoperatively, patients were usually immobilized in a rigid cervical collar (Miami J) for 6–12 weeks. nonsurgically managed patients were treated with rigid cervical collar immobilization for 12 weeks. Management strategies followed institutional practice; all AO type B or C fractures were considered for surgery, with a proposed treatment plan (surgery or conservative treatment) after thorough clinical assessment and internal discussion.

Patients with cervical spinal cord injury (cSCI) were managed in the neurointensive care unit targeting a mean arterial pressure (MAP) of 85 mmHg (range 80–90 mmHg) for 5–7 days, and patients with residual neurological deficits were routinely referred for specialized rehabilitation if deemed appropriate by an ambulatory rehabilitation team.

### Long-term follow-up and outcome measures

The primary outcome was the long-term neck-related disability measured by NDI, comprising 10 items with a total score ranging from 0 to 50 points. Scores were reported as percentages ranging from 0% to 100% [26].

Secondary outcomes included living status, work status, cSCI according to AIS, radiculopathy, neck and arm pain intensity measured by NRS, and radiological outcome on cervical CT. Global functional status was assessed using the Karnofsky Performance Status (KPS), a scale ranging from 0 to 100 that reflects overall functional independence. Although originally developed for oncology patients, KPS is also used as a generic measure of functional performance across medical and surgical populations [34].

Radiculopathy, defined as any residual dermatomal arm pain, was only scored for patients without cSCI at admission (AIS E), as reliable assessment is limited in patients with impaired sensory or motor function due to spinal cord injury. Neuropathic pain was not quantified for patients with cSCI (AIS A–D).

Study-specific CT scans were compared with the last CT scan performed for each patient and were routinely reviewed blinded by a senior neuroradiologist. Furthermore, all scans were reviewed by 2 experienced neurosurgeons. Cases with discrepant or uncertain findings were rereviewed jointly and resolved by consensus discussion; formal inter and intrarater reliability analyses were not performed. The following domains were assessed: bony fusion (yes/no/partial), pseudoarthrosis (yes/no), implant-related changes including fracture or pull-out of hardware (yes/no), and changes in cervical alignment (ie, increased fracture gap, increased angulation—yes/no). Pseudoarthrosis was defined as persistent fracture lines with no bridging trabecular bone and presence of sclerotic margins on CT performed ≥3 months after injury.

### Statistical analyses

Descriptive statistics summarized baseline characteristics. Continuous variables are presented as mean ± standard deviation (SD) or median with interquartile range (IQR), as appropriate, and categorical variables as counts and percentages. Descriptive group comparisons were performed using nonparametric methods, including the Mann–Whitney U and Kruskal–Wallis tests, given the non-normal distribution of the data. Paired comparisons were performed using the Wilcoxon signed-rank test. Fisher’s exact test was used for comparison of categorical variables.

Given the elderly and comorbid nature of the cohort, nonapplicable items in the Neck Disability Index (NDI) were treated as missing, and total NDI scores were normalized to the number of completed items, expressed as percent. This approach has previously been shown to preserve the validity of NDI scoring in elderly and retired populations [26]. Patients were stratified into 3 age groups (<60, 60–69, and ≥70 years). For patients who sustained more than 1 cervical spine fracture during the study period, long-term clinical outcomes were assessed once at the patient level. The final follow-up assessment therefore reflected cumulative functional status at follow-up. Age-group comparisons of NDI were performed using the Kruskal–Wallis test. Confidence intervals for median NDI values were estimated using bootstrap resampling (5,000 samples).

Since follow-up was not performed at predefined time points after injury, but varied according to the time of injury and long-term follow-up date, follow-up duration was analyzed using Spearman’s rho and additional nonparametric methods. Patients were additionally stratified into 3 follow-up intervals (0.4–3.9, 4–6.9, and 7–12.4 years), and similar nonparametric methods were applied. Because 10 patients (10%) had follow-up shorter than 1 year, sensitivity analyses were performed after excluding these patients.

Missing data for Karnofsky Performance Status (4 cases, 4%) and study-specific CT (2 cases, 2%) were limited and considered unlikely to be related to patient characteristics or outcomes, and therefore assumed not to meaningfully influence the results. No imputation or formal sensitivity analyses were performed.

All statistical analyses were performed using SPSS version 30 (IBM Corp.) and R (R Foundation for Statistical Computing). Given the exploratory nature of the study, p-values are reported and interpreted descriptively. All tests were 2-sided. No a priori power calculation was performed, as this was a descriptive survivor cohort study including all eligible long-term survivors from a rare clinical population. As a sensitivity analysis, we estimated the detectable effect size for the exploratory comparison of NDI between surgically and nonsurgically managed patients in the prospectively captured 2015–2020 subcohort. With 48 surgically and 21 nonsurgically managed patients, a 2-sided alpha of 0.05, and 80% power, the study would be able to detect a large standardized between-group difference of approximately Cohen´s d = 0.75. Assuming an SD of 18.3 percentage points for NDI, this corresponds to an absolute difference of approximately 14 percentage points in NDI.

## Results

### Study population and follow-up

**Fig. 1:**
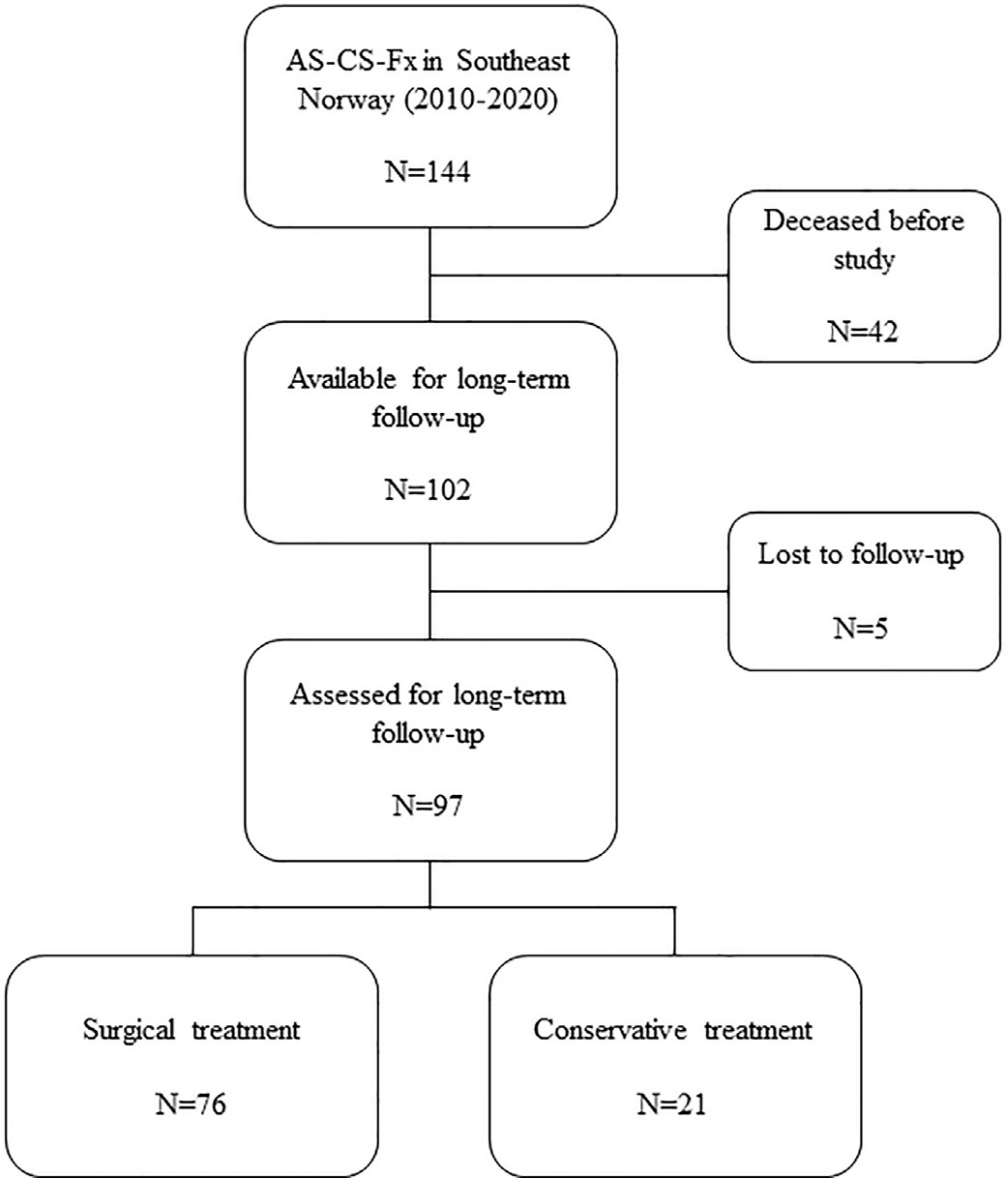
Flow diagram of the AS-CS-Fx study population, illustrating patient inclusion, follow-up, and treatment allocations. AS, ankylosing spondylitis; CS-Fx, cervical spine fracture.

Between January 2010 and December 2020, we identified 144 patients, of whom 7 sustained ≥2 cervical spine fractures (CS-Fx) as separate injuries. At the time of study invitation, 102 of 144 identified patients were alive and eligible for long-term follow-up. The remaining 42 patients had died before the invitation. Of the eligible 102 survivors, 5 declined participation or did not respond, leaving 97 patients with available long-term follow-up data (Fig. 1). Among these 97 patients, 4 sustained 2 CS-Fx, and 1 sustained 3 CS-Fx in separate injury events. Median follow-up time was 4.2 years (IQR 4.8, range 0.4–12.4 years). Baseline characteristics of the long-term follow-up cohort are presented in Table 1.

**Table 1:** Baseline patient characteristics of the study population 2010-2020 (n = 97).

Variable	Total = 97 patients
Age, y (median, IQR)	66 (58-73)
Male sex, n (%)	86 (89)
Preinjury ASA class, n (%)	
II	34 (35)
III	61 (63)
IV	2 (2)
Preinjury living	
Independent, n (%)	91 (94)
Occupational status, n (%)	
Working	27 (28)
Retired	46 (47)
Disabled	20 (21)
Unknown	4 (4)
Cervical fracture level, n (%)	
C0–C2	5 (5)
Odontoid	5 (5)
C2/C3	2 (2)
C3/C4	7 (7)
C4/C5	6 (6)
C5/C6	27 (28)
C6/C7	37 (38)
C7/Th1	8 (9)
AIS at admission, n (%)	
A	2 (2)
B	2 (2)
C	3 (3)
D	7 (8)
E	83 (85)
sEDH, n (%)	
Unknown, no MRI	8 (12)
With mass-effect on S.C	9 (13)
Without mass-effect on S.C	9 (13)
Radiculopathy, n (%)	28 (34)
Polytrauma, n (%)	31 (32)
Thoracolumbar Fx	17 (18)
Surgical treatment, n (%)	76 (78)
Anterior-only	5 (7)
Posterior-only	49 (64)
Circumferential	22 (29)

ASA, American Society of Anesthesiologists; AIS, American Spinal Injury Association Impairment Scale; IQR, interquartile range; MRI, magnetic resonance imaging; S.C, spinal cord; sEDH, spinal epidural hematoma.

No association was observed between follow-up duration and long-term disability outcomes; NDI scores did not differ across follow-up duration groups (0.4–3.9, 4–6.9, and >7 years; Kruskal–Wallis H = 1.79, p = .41), and no monotonic association was observed when follow-up duration was analyzed as a continuous variable (Spearman’s ρ = −0.11, 95% CI −0.31 to 0.10; p = .284).

Ten patients (10%) had follow-up shorter than 1 year. Sensitivity analyses restricted to patients with follow-up ≥1 year (n = 87) yielded similar results, with no clear monotonic association between follow-up duration and NDI (Spearman’s ρ = −0.17, 95% CI −0.37 to 0.05; p = .108).

In the 2015–2020 subcohort (n = 69), the median follow-up time was 3.0 years (IQR 1.6–4.7; range 0.4–6.9 years). Follow-up duration did not differ significantly between surgically and nonsurgically treated patients (Mann–Whitney U test, p = .67).

### Management

Among the 97 patients with long-term follow-up, 76 (78%) were treated surgically and 21 (22%) nonoperatively. Posterior fixation was the most common surgical approach, followed by circumferential and anterior-only procedures (Table 1).

Nonoperative management was primarily reserved for patients with substantial medical comorbidity, frailty (typically ASA Physical Status 4), or selected fracture patterns considered relatively stable, including isolated spinous process fractures and selected upper cervical fractures. Anterior-only procedures were uncommon and were generally performed in patients in whom prolonged prone positioning or extensive posterior surgery was considered poorly tolerated because of patient-related factors and comorbidity.

Because nonoperative cases were incompletely captured prior to 2015, comparative analyses between surgically and nonsurgically managed patients were restricted to those included from 2015 onward, when prospective registration ensured complete case identification. Baseline characteristics of surgically and nonoperatively managed patients treated between 2015 and 2020 are shown in Table 2.

**Table 2:** Baseline characteristics in the surgical and nonsurgical group (2015–2020).

Variable	Surgery (n = 48)	Nonsurgical (n = 21)
Age, y (median, IQR)	65.5 (58-73)	66 (54-72)
Male sex, n (%)	42 (88)	19 (90)
Preinjury living (independent), n (%)	46 (96)	18 (86)
Working, n (%)	19 (40)	3 (14)
Unknown, n (%)	0 (0)	4 (19)
Retired, n (%)	24 (50)	9 (43)
Preinjury ASA, n (%)		
II	22 (46)	7 (33)
III	26 (54)	12 (57)
IV	0 (0)	2 (10)
AO fracture type (B or C), n (%)	46 (96)	8 (38)
Spinal cord injury (AIS A–D), n (%)	9 (19)	0 (0)
sEDH (with mass-effect), n (%)	9 (19)	0 (0)
Radiculopathy at presentation*, n (%)	12 (31)	3 (14)
Polytrauma	17 (35)	9 (43)

ASA, American Society of Anesthesiologists; AIS, American Spinal Injury Association Impairment Scale; AO, Arbeitsgemeinschaft für Osteosynthesefragen fracture classification; IQR, interquartile range; sEDH, spinal epidural hematom.

⁎ Radiculopathy only scored for patients with AIS E.

### Primary outcome: Neck Disability Index (NDI)

In the total long-term follow-up cohort (n = 97), the median NDI was 16% (IQR 6–30; 95% CI 10–20), corresponding to mild residual disability in the majority of patients (Table 3). Restricting the analysis to patients included between 2015 and 2020, for whom both surgical and nonsurgical cases were completely captured (n = 69), the median NDI was 18% (IQR 6–32; 95% CI 10–24).

**Table 3:** Primary and secondary outcome variables for the long-term cohort (n = 97).

Outcome at follow-up	n/N (%) or median (IQR)
NDI (%)	16 (6–30)
Living independently	78/97 (80)
Living with assistance	18/97 (19)
Institutionalized	1/97 (1)
Employment maintained after injury	22/27 (81)
Still working at last follow-up	15/27 (56)
Karnofsky performance score	80 (70–90)
Residual cSCI (AIS A-D)	7/97 (7)
Radiculopathy at follow-up*	10/83 (12)
Radicular pain NRS (among pt with radiculopathy)	1 (1–2)
Neck pain at follow-up	61/97 (63)
Neck pain NRS (among pt with neck pain)	2 (1–5)

AIS, American Spinal Injury Association Impairment Scale; cSCI, cervical spinal cord injury; IQR, interquartile range; NDI, Neck Disability Index; NRS, Numeric Rating Scale; Pt, patients.

⁎ Radiculopathy only scored for patients with AIS E.

**Fig. 2:**
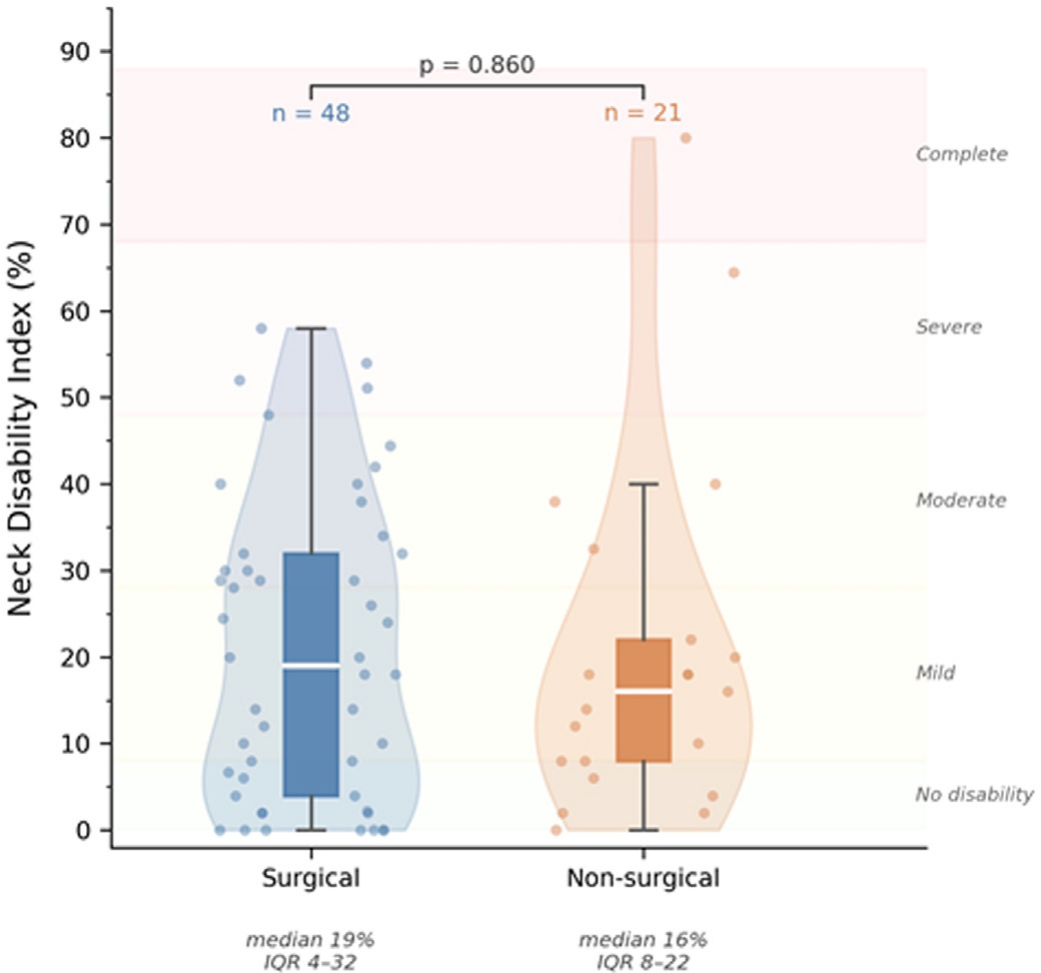
Distribution of Neck Disability Index (NDI) scores at follow-up in surgically and nonsurgically treated patients from the 2015–2020 subcohort. NDI, Neck Disability Index.

Within the 2015–2020 subcohort, median NDI was 19% (IQR 4–32; 95% CI 10–29) in surgically treated survivors and 16% (IQR 7–27; 95% CI 8–21) in nonsurgically managed survivors (Mann–Whitney U test, p = .855) (Fig. 2). The observed between-group difference in NDI was small relative to the estimated detectable effect size in the sensitivity power analysis. This comparison was unadjusted and exploratory.

Sensitivity analyses excluding patients with follow-up <1 year (n = 10) showed similar results (median NDI 16%, IQR 6–31).

Five patients sustained more than 1 cervical spine fracture event. Their median NDI was 20% (IQR 13–56), compared with 15% (IQR 5–30) among patients with a single fracture event.

**Fig. 3:**
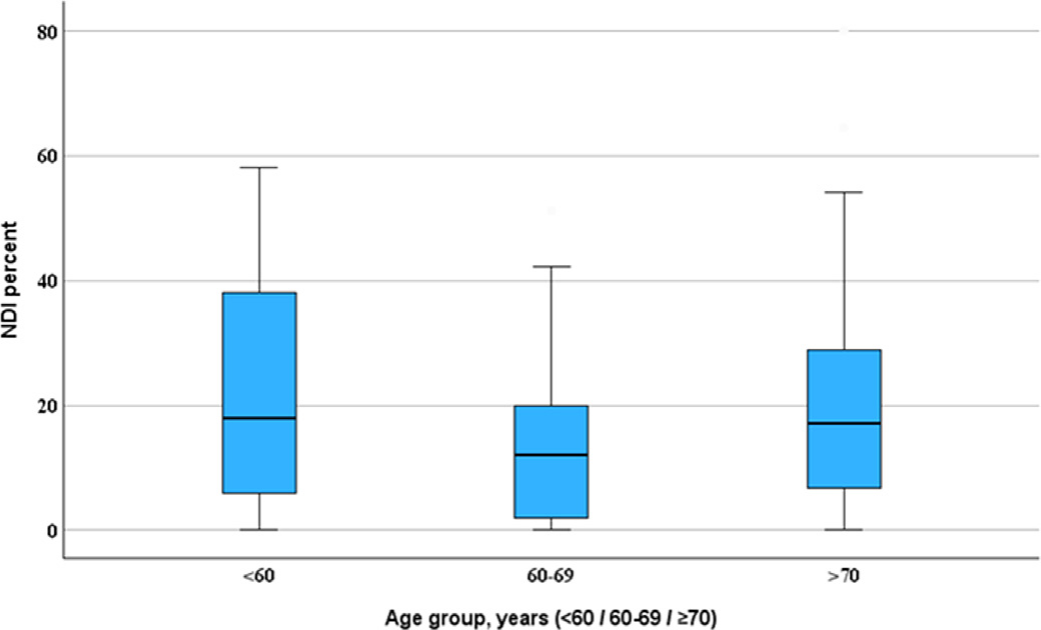
Distribution of Neck Disability Index (NDI) scores across age groups. NDI, Neck Disability Index.

Median long-term NDI did not differ significantly across age groups (<60, 60–69, ≥70 years; Kruskal–Wallis H = 2.12, p = .346) (Fig. 3). Residual spinal cord injury at follow-up (AIS A–D vs. E) was associated with higher long-term disability, as measured by NDI (Mann–Whitney U test, exact p = .028), but not with the presence of neck pain at follow-up (Fisher’s exact test, p = .42).

### Secondary outcomes

#### Living status and Karnofsky Performance Status (KPS)

Prior to injury, 91 of 97 patients (94%) lived independently. At long-term follow-up, 78 patients (80%) remained living independently, 18 (19%) required assistance at home, and 1 patient (1%) was institutionalized; no patient transitioned directly from independent living to institutional care (Table 3). KPS was available for 93 of 97 patients at follow-up, with a median score of 80 (IQR 70–90).

### Working status

Of the 27 patients employed prior to injury, 22 (81%) remained employed for several years after the injury or until retirement. At follow-up, 15 patients were still in active employment. Only 1 patient became permanently work-disabled as a direct consequence of the injury (Table 3).

### Cervical spinal cord injury (cSCI)

**Fig. 4:**
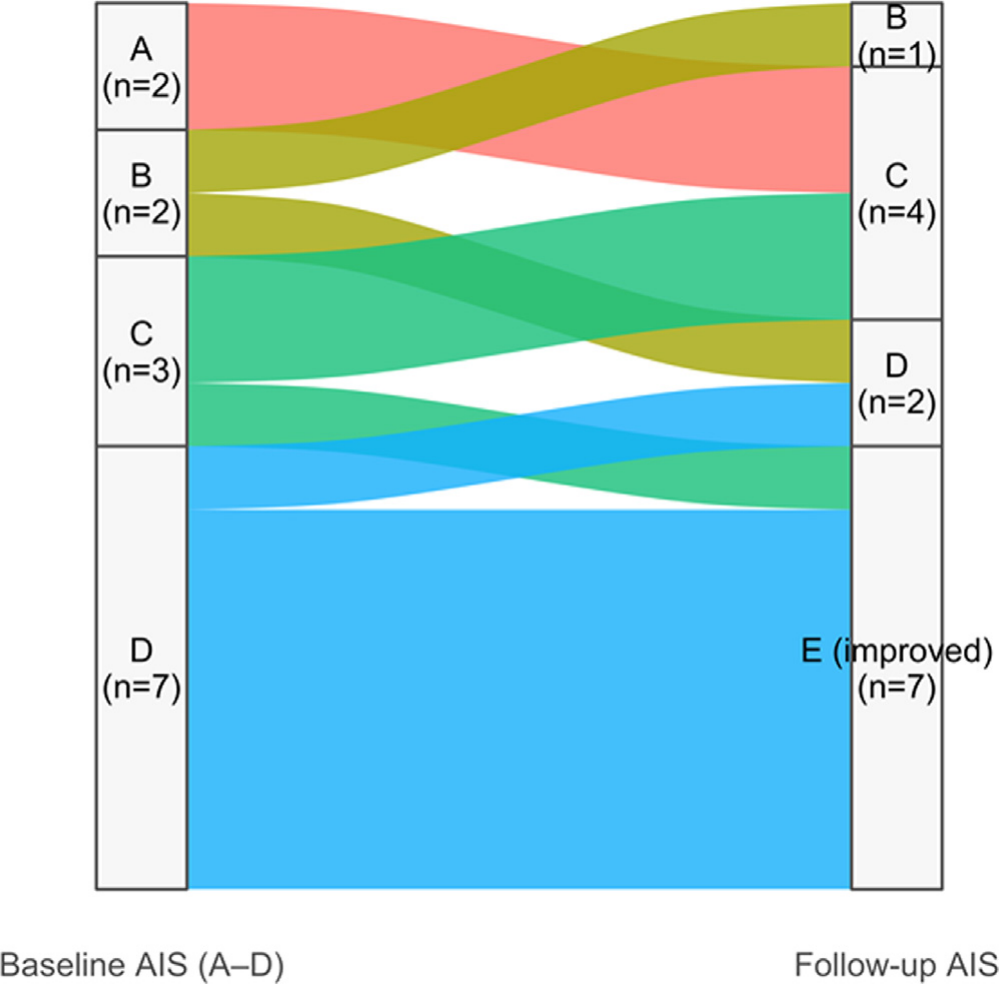
Changes in neurological status from baseline to follow-up, illustrated by transitions in AIS grade. The width of each band represents the number of patients. AIS, American Spinal Injury Association Impairment Scale.

At admission, 14 of 97 patients (14%) presented with cervical spinal cord injury (AIS A–D). At long-term follow-up, 7 of these patients (50%) had improved to AIS E (no residual spinal cord impairment), while 7 had persistent neurological impairment. No patient deteriorated neurologically during follow-up. Overall, improvement by at least 1 AIS grade was observed in 10 of the 14 patients with cervical spinal cord injury (Fig. 4). Comparison of admission and long-term follow-up AIS scores demonstrated a significant overall improvement in neurological status (Wilcoxon signed-rank test, Z = −2.89, p = .004).

### Radiculopathy (dermatomal arm pain)

Radiculopathy was present at injury in 28 of 83 patients without cervical spinal cord injury (AIS E) (34%). At long-term follow-up, persistent radiculopathy was observed in 10 of 83 patients (12%), corresponding to resolution of radicular symptoms in 18 of 28 patients (64%). Among patients with persistent radiculopathy, median arm pain intensity was 1 on the NRS scale (IQR 1–2) (Table 3).

### Neck pain

At long-term follow-up, neck pain (NRS > 0) was reported by 61 of 97 patients (63%), while 36 patients (37%) reported no neck pain. Among patients reporting pain, median neck pain intensity was 2 on the NRS scale (IQR 1–5) (Table 3).

### Radiological outcome

Cervical CT performed at follow-up was available in 95 of 97 patients (98%) and demonstrated solid bony fusion in 91 patients (96%). Implant position changes, limited to partial screw pull-out, were detected in 7 patients, 3 of whom underwent elective removal of osteosynthesis material after confirmation of solid fusion on long-term CT. Partial fusion of the C1 ring was identified in 1 patient (1%) (Table 4).

**Table 4:** Long-term radiological outcome.

Radiological outcome (CT)	n/N (%)
CT available	95/97 (98)
Solid bony fusion	91/95 (96)
Partial fusion (C1 arch)	1/95 (1)
Fibrous nonunion (odontoid)	1/95 (1)
Pseudoarthrosis*	3/95 (3)
Implant position change	7/95 (7)
Changed cervical alignment	0/95 (0)

CT, computed tomography.

⁎ Pseudoarthrosis included 2 isolated spinous process fractures and 1 odontoid fracture.

## Discussion

To our knowledge, the present study represents one of the most comprehensive long-term evaluations of surviving patients with cervical spine fractures in ankylosing spondylitis, combining standardized patient-reported outcome measures with systematic CT-based radiological assessment. Despite advanced age and the severe nature of AS-associated cervical spine fractures, most surviving patients reported low levels of neck-related disability and pain, preserved global function, and high rates of radiological healing at long-term follow-up.

### Neck disability and neck pain

Although the Neck Disability Index (NDI) was the primary outcome measure, it is discussed together with neck pain intensity, as these outcomes are commonly reported in combination in previous studies. NDI scores are often interpreted according to severity categories, where values in the range of approximately 10%–28% are commonly considered to reflect mild residual disability. At a median follow-up of 4.2 years, the median NDI in the present cohort (n = 97) was 16% (IQR 6–30), corresponding to mild residual disability in most patients. Although neck pain was frequently reported, its intensity was low, with a median NRS score of 2 (IQR 1–5), suggesting limited long-term clinical impact.

These findings are broadly consistent with prior reports from surgically treated AS-associated cervical fracture cohorts. Alhashash et al. [11] reported improvement in mean NDI from approximately 34 preoperatively to 15 at follow-up, along with a reduction in VAS neck pain from 7.3 to 3.2 in a 2-center surgical series. Robinson et al. similarly reported low long-term neck and arm pain scores following posterior fixation, with continued improvement over time, consistent with findings from smaller surgical cohorts demonstrating early postoperative pain reduction [18,35].

Previous studies have largely focused on surgically treated patients with short- to midterm follow-up. In contrast, the present study includes both surgically and nonsurgically managed patients with longer follow-up, allowing assessment of sustained functional outcome beyond the early recovery phase. In an unadjusted exploratory comparison within the prospectively captured 2015–2020 subcohort, median NDI was 19% in surgically treated and 16% in nonsurgically managed survivors. Because treatment allocation reflected fracture morphology, neurological status, comorbidity, and frailty, this comparison should not be interpreted as evidence of equivalence or comparative effectiveness (Table 2).

In a recent study on nonfracture AS patients, the median NDI score was 17.5 (IQR 11.0–26.25) in 30 patients [36]. In the present cohort, the median long-term NDI was only modestly higher than values reported in a large U.S. general population survey (median 13, IQR 6–22) [37]. Reference values from nonfracture AS cohorts and population-based samples provide context for interpreting the magnitude of NDI scores, but they do not allow inference regarding recovery from the fracture event. The present cohort lacked preinjury NDI, BASDAI, or BASFI measurements, and direct adjustment for differences between AS-CS-Fx survivors, nonfracture AS patients, and general population samples was not possible. Accordingly, the median NDI of 16% should be interpreted as the level of neck-related disability among long-term survivors, not as a direct estimate of fracture-related disability. In AS, pain and disability may also be influenced by inflammatory mechanisms rather than purely mechanical factors related to fracture morphology, instability, or fusion status, which further complicates attribution of long-term NDI solely to the fracture event.

Neither age nor follow-up duration was associated with long-term NDI, suggesting that residual disability among survivors did not vary substantially across the observed follow-up intervals.

### Living, working, and Karnofsky Performance Status

Global functional outcome was preserved in most survivors. A median Karnofsky Performance Status (KPS) score of 80 (interquartile range 70–90) indicates that the majority of patients were able to perform normal activities with only minor limitations. In line with this, 80% of patients lived independently at long-term follow-up, while institutionalization was rare.

Among patients employed prior to injury, most remained employed for several years or until retirement. Continued employment among previously working patients supports the observation of preserved long-term functional capacity, an outcome infrequently reported in cohorts of patients with AS-associated cervical spine fractures [38].

### Neurological status

Persistent neurological impairment was associated with higher long-term disability. Patients with residual spinal cord injury at follow-up had significantly higher NDI scores than neurologically intact patients, in line with previous studies demonstrating neurological injury as a key determinant of functional outcome after cervical spine trauma [23,[39], [40], [41]].

Encouragingly, half of the patients with SCI at presentation recovered to AIS E, and 71% improved by at least 1 AIS grade during follow-up, broadly consistent with previous AS-CS-Fx cohorts reporting neurological improvement in a substantial proportion of patients [3,35,42,43]. Despite its impact on disability, persistent spinal cord injury was not associated with the presence of neck pain at follow-up, highlighting the heterogeneous relationship between neurological impairment and axial pain after cervical spine trauma [44,45].

Radiculopathy resolved in the majority of patients. Although one-third of patients presented with radicular symptoms at injury, only a minority reported persistent radiculopathy at long-term follow-up, and residual arm pain intensity was low (median NRS 1, IQR 1–2). While resolution rates appear somewhat lower than those reported in non-AS cervical fracture populations [46], the low pain intensity suggests limited long-term clinical impact.

### Radiological outcome and fusion

Radiological healing was achieved in most patients, with solid bony fusion demonstrated in 96% of assessed cases, comparable to fusion rates reported in smaller surgical series, often approaching 100% [18,35,47,48]. All cases of pseudoarthrosis or nonunion occurred in conservatively managed spinous process or odontoid fractures, fracture types known to be associated with fibrous healing or nonunion [49]. Minor implant-related changes were observed in a small proportion of patients and were limited to partial screw pullout. In most cases, these changes were already evident on routine 12-week postoperative cervical CT. These findings suggest that clinically apparent late radiological changes were uncommon among survivors in this cohort.

Because early mortality after cervical spine fractures in ankylosing spondylitis is strongly influenced by age, frailty, and comorbidity, mortality was addressed separately in a prior population-based analysis and was not included as an outcome in the present survivor-focused study [50].

### Strengths and limitations

This study has several limitations. Because the study was designed as a cross-sectional long-term follow-up of survivors, the findings apply only to patients who were alive and eligible for long-term assessment. They should therefore not be interpreted as describing outcomes in the full AS-CS-Fx source population. Although the study represents one of the largest comprehensive long-term cohorts of patients with ankylosing spondylitis-associated cervical spine fractures, subgroup analyses were based on relatively modest sample sizes. Accordingly, the exploratory surgical versus nonsurgical comparison was underpowered to detect smaller but potentially clinically relevant between-group differences, and nonsignificant findings should therefore be interpreted cautiously. There were substantial baseline differences between surgically and nonsurgically treated patients, and treatment allocation was not randomized but reflected clinician judgment based on fracture morphology, neurological status, comorbidity, and frailty, limiting causal interpretation of between-group comparisons. Outcomes were assessed at a single long-term time point without preinjury patient-reported outcome measures, limiting evaluation of individual longitudinal trajectories. Although a minority of patients were assessed before 12 months, sensitivity analyses excluding these patients yielded similar estimates of long-term disability.

The main purpose of follow-up radiology beyond the standard 3-month assessment was to detect possible clinically meaningful radiological changes. Study-specific CT scans were systematically reviewed using predefined categorical criteria by a blinded senior neuroradiologist and 2 experienced neurosurgeons. Cases with discrepant or uncertain findings were rereviewed jointly and resolved by consensus discussion. Formal inter and intrarater reliability analyses were not performed, as the radiological assessment was designed as a pragmatic clinical review rather than a dedicated imaging reliability study. Nevertheless, formal assessment of observer agreement would have strengthened the methodological rigor of the radiological evaluation.

Patient-reported outcome measures such as the Neck Disability Index and Numeric Rating Scale are not disease-specific instruments and do not fully capture ankylosing spondylitis–related disease activity, including inflammatory burden, spinal stiffness, fatigue, or extra-articular manifestations. Nevertheless, these generic PROMs remain clinically meaningful, and provide a pragmatic estimate of neck-related functional status in elderly and comorbid survivors. Another limitation is that the diagnosis of ankylosing spondylitis could not be confirmed with equal certainty in all patients. Some patients may have had other axial spondyloarthropathies or ankylosing spinal disorders, and in selected cases inclusion was based primarily on characteristic CT findings of spinal ankylosis. However, the clinically relevant common denominator was an ankylosed cervical spine, which represents the principal biomechanical substrate for unstable fracture patterns and complex treatment decisions. Thus, although some diagnostic heterogeneity is possible, the cohort likely reflects the clinically relevant population of patients with ankylosed cervical spine fractures.

Strengths of the study include a survivor cohort nested within a population-based cervical spine fracture registry from a centralized neurotrauma center, long-term follow-up exceeding 4 years, a high response rate among eligible survivors, and systematic assessment of both clinical and radiological outcomes using predefined CT-based criteria. The inclusion of both surgically and nonsurgically managed patients from a prospectively captured subcohort further enhances the clinical relevance of the findings within a centralized neurotrauma setting.

## Conclusion

Among survivors of ankylosing spondylitis-associated cervical spine fractures, long-term neck-related disability and pain were generally low, most patients remained independent, and CT demonstrated bony fusion in most assessed patients. These findings characterize long-term survivor outcomes and should not be extrapolated directly to the entire AS-CS-Fx source population. Persistent neurological impairment was associated with higher disability. Unadjusted exploratory comparisons between surgically and nonsurgically managed survivors did not demonstrate a large between-group difference in NDI, but these analyses were limited by small sample size, baseline imbalance, and surgeon-directed treatment allocation. These findings may help inform both clinicians and patients regarding expected ranges of clinical outcome in long-term survivors of ankylosing spondylitis-associated cervical spine fractures.

## Funding

This research received no external funding.

## Ethics approval

Approved by the Oslo University Hospital Data Protection Officer (DPO), approval numbers 18/24228 and 2014/12304. The Regional Committee for Medical and Health Research Ethics reviewed the project and classified the study as a quality control project outside the scope of the Norwegian Health Research Act, for which formal REC approval was not required (ref. no. 2019/133).

## Declaration of generative AI and AI-assisted technologies in the writing process

During the preparation of this manuscript, the authors used an AI-based language model (ChatGPT, OpenAI) for minor language editing. The authors reviewed and revised the text as needed and take full responsibility for the content of the manuscript.

## CRediT authorship contribution statement

**Pål Nicolay Fougner Rydning:** Conceptualization, Data curation, Formal analysis, Investigation, Methodology, Software, Validation, Visualization, Writing – original draft. **Hege Linnerud:** Data curation, Investigation, Supervision, Writing – review & editing. **Vidar Stenset:** Project administration, Resources, Supervision. **Jalal Mirzamohammadi:** Supervision. **Dag Ferner Netteland:** Supervision, Validation. **Tor Brommeland:** Investigation, Supervision, Validation, Writing – review & editing. **Magnus Evjensvold:** Investigation, Validation. **Carl-Johan Henie Sogn:** Data curation, Formal analysis, Software, Visualization, Writing – review & editing. **Eirik Helseth:** Conceptualization, Methodology, Project administration, Supervision, Writing – review & editing. **Markus Karl Hermann Wiedmann:** Conceptualization, Methodology, Supervision, Writing – review & editing.

## Declaration of competing interests

The authors declare that they have no competing interests relevant to this work.
